# A Group Videoconferencing Intervention (C@nnected) to Improve Maternal Sensitivity: Protocol for a Randomized Feasibility Trial

**DOI:** 10.2196/35881

**Published:** 2022-08-15

**Authors:** Victoria Binda, Marcia Olhaberry, Carla Castañon, Constanza Abarca, Catalina Caamaño

**Affiliations:** 1 Deparment of Family Medicine Medicine Faculty Pontificia Universidad Católica de Chile Santiago Chile; 2 School of Psychology Social Sciences Faculty Pontificia Universidad Católica de Chile Santiago Chile

**Keywords:** maternal sensitivity, group intervention, primary care, eHealth, responsive caregiving, videoconferencing, Early childhood, caregiver, eHealth intervention, health intervention, parenting, children, peer-support

## Abstract

**Background:**

Early childhood development is highly dependent on the sensitive care provided by caregivers, and interventions focused on supporting parents to improve their sensitivity have shown to be effective. The COVID-19 pandemic has had a significant impact on mental health, with pregnant women and mothers of infants being an especially vulnerable group and maternal sensitivity particularly affected. However, access to face-to-face interventions is restricted; thus, it is important to have remote interventions to support this group of mothers.

**Objective:**

The objective of this study is to evaluate the feasibility and acceptability of C@nnected, a group videoconferencing intervention to improve maternal sensitivity aimed at mother-infant dyads attending primary health care centers in vulnerable areas of Santiago, Chile.

**Methods:**

This is a randomized feasibility single-masked (outcome assessor) study with a qualitative component. It will involve a block randomization procedure to generate a 3:2 allocation ratio (with more people allocated to the intervention arm). The intervention consists of 4 group videoconferencing sessions adapted from a face-to-face intervention with proven effectiveness. The control group will receive treatment as usual, along with educational brochures. The feasibility and acceptability of this study will be quantitatively and qualitatively assessed. Changes in clinical outcomes relating to maternal sensitivity, depressive symptoms, postpartum maternal attachment, and infant socioemotional development will also be evaluated.

**Results:**

We finished adapting the face-to-face intervention to the videoconferencing format in July 2021. The study began recruitment in August 2021, and enrollment is expected to end in August 2022, with final study results expected in December 2022.

**Conclusions:**

This study will contribute evidence for the use of eHealth interventions to promote maternal sensitivity. It will also inform the design and implementation of a future randomized clinical trial.

**Trial Registration:**

ClinicalTrials.gov NCT04904861; https://clinicaltrials.gov/ct2/show/NCT04904861

**International Registered Report Identifier (IRRID):**

DERR1-10.2196/35881

## Introduction

### Background and Rationale

Ensuring adequate early childhood development is necessary for countries to move forward in an equitable and sustainable way; therefore, interventions to support early childhood development are essential to realize the United Nations Sustainable Development Goals [1]. The experience that best facilitates adequate child development is responsive caregiving provided by parents or primary caregivers [2], and one of the most relevant predictors of child development is the quality of the mother-child interaction [3]. The more sensitive, responsive, attentive, and cognitively stimulating the mother is, the better the results in her child. The main risk factors associated with the presence of low maternal sensitivity are maternal depression [4-6], low socioeconomic status, and other psychosocial risk factors [7], which are mediated by the high levels of stress experienced by these families [8].

Before 2020, mental health disorders were leading causes of global health-related burdens. With the emergence of the COVID-19 pandemic, mental health disorders have greatly increased across the world [9], especially in high-risk populations [10]. Pregnancy and postpartum are periods of special vulnerability for mental health. There are short- and long-term negative consequences to the physical, cognitive, and psychological development of children associated with prenatal and postpartum stress, anxiety, and depression [11], which are mainly due to a decrease in the quality of mother-child interactions. A recent Canadian study [12] carried out in pregnant women and those with children under 1 year of age showed an increase in anxiety symptoms from 29% to 72% after the COVID-19 pandemic and postpartum depression symptoms from 15% to 47%. The risk of mental health problems in pregnancy and the first year postpartum in the context of the pandemic has increased due to concerns related to the well-being of the child and aggravated by the consequences of preventive measures such as confinement, physical distance, reduced health checks, and difficulty obtaining support from the usual networks [13]. Moreover, these mental health problems are present at a greater extent in women of low socioeconomic status. Countries are recommended to implement mitigation strategies to reduce the mental health burden imposed by COVID-19, considering their local context and vulnerable populations [9].

For mothers to provide the responsive caregiving that their children need, they must have adequate mental health and a support network. Early interventions focused on supporting parents in providing responsive caregiving have proven to be effective [14-16]. Health services provide a critical starting point for these interventions, given their reach to pregnant women, families, and young children [16,17]. Thus, it is imperative to implement supportive interventions for pregnant and postpartum women that promote adequate sensitivity toward their children.

Global lockdown measures have disrupted routine health care for non–COVID-19 patients, so telemedicine has been escalated to reduce the risks of disease transmission [18]. Electronic mental health care is one of the most widely offered methods of health care, with evidence of applicability and efficacy in a wide range of formats [19]. A systematic review showed that group video conferencing interventions are feasible, improve access to health care, and have results similar to those obtained in face-to-face groups [20]. There is an interest in retaining or further incorporating virtual components devised in response to the COVID-19 pandemic into the standard delivery of interventions in the future [21]. In this context, it is important to implement remote interventions in primary health care (PHC) for mothers of children under 1 year of age that promote responsive caregiving.

### Chilean Context

Chile is a Latin American country that presents an important burden of mental health problems. The World Health Organization places Chile among the countries with the highest burden of morbidity from psychiatric diseases [22], and it has the world’s highest rates of postpartum depression [23,24] and preschool mental health problems [25]. Despite this, only 38.5% of those diagnosed receive some type of mental health service [22]. Chile has a comprehensive protection system for children from the prenatal period to 4 years of age known as Chile Crece Contigo (translated as “Chile Grows With You”) that aims to help all children reach their full potential for development through universal and targeted support services [26]; however, no specific interventions are offered to promote maternal sensitivity.

In 2 previous research projects, we developed and evaluated a face-to-face group intervention to promote maternal sensitivity during the child's first year of life [27]. The results, which will be published soon, showed positive effects on maternal sensitivity in mother-infant dyads attending PHC centers but low adherence rates. We also found a significant reduction in maternal depression symptoms and infant socioemotional development difficulties of resident dyads at Chilean female penitentiary centers [28].

### Objective

The primary aim of this paper is to report on the protocol comprising a pilot randomized feasibility trial to evaluate the feasibility and acceptability of C@nnected, a group videoconferencing intervention that aims to improve maternal sensitivity in mother-infant dyads attending PHC centers in Chile. Along with detailing the intervention, this paper provides an account of the plan to collect both quantitative and qualitative measurements of outcomes.

The secondary aims are to (1) to estimate the effect size in clinical outcomes between the groups after the intervention in terms of maternal sensitivity, postpartum depressive symptoms, postpartum attachment, and socioemotional development; and (2) identify the key parameters for the implementation and evaluation of the intervention, which will enable the design of an effectiveness study in the future.

## Methods

### Trial Design

This is a data analyst–blind, superiority, pilot feasibility study with a mixed design. The quantitative component is a 2-arm randomized parallel group of 50 mothers of infants aged 6-12 months who receive health care in 2 PHC centers in Santiago, Chile. Mothers will be randomized into the intervention or control group in a to 3:2 ratio (with more people allocated to the intervention arm). Both groups will receive the usual treatment and educational brochures provided by the PHC center, with the intervention group also receiving the C@nnected videoconferencing intervention. The qualitative component will consist of semistructured interviews with intervention providers to identify possible improvements and core components of the intervention as well as focus groups with mothers participating in the intervention to evaluate user acceptability and satisfaction with the implementation.

**Figure 1:**
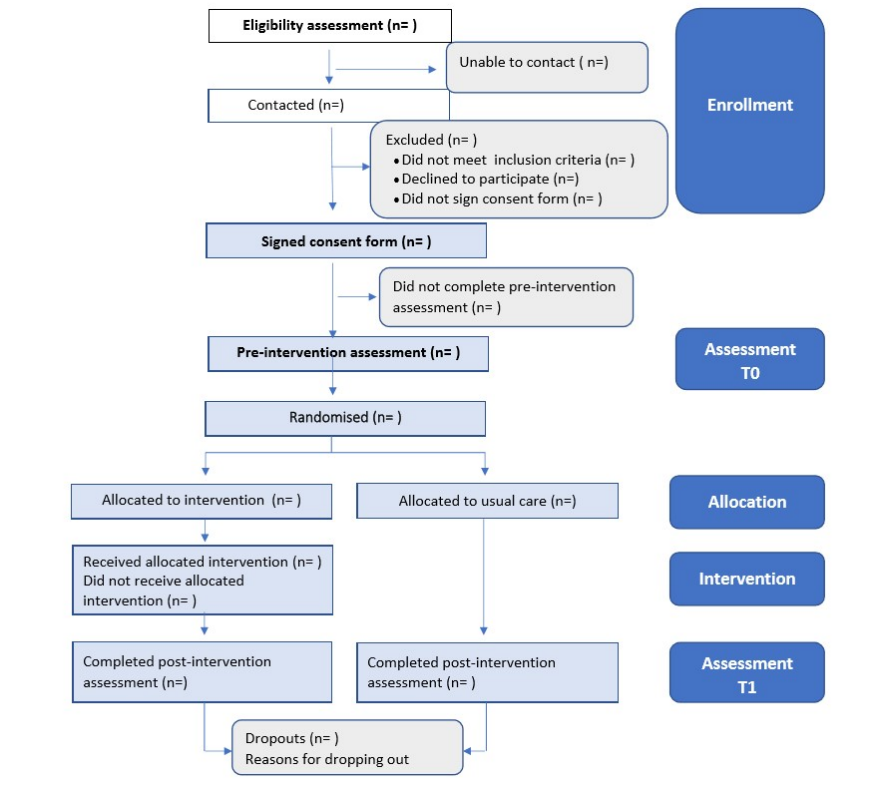
Flowchart of participants. T0: preintervention assessment; T1: postintervention assessment.

This type of study is suggested by the literature as an initial requirement for the implementation of an innovative intervention before applying it on a larger scale. It is also recommended to use quantitative and qualitative methodologies to evaluate feasibility and acceptability [29]. This clinical trial protocol follows the SPIRIT (Standard Protocol Items: Recommendations for Interventional Trials) and [30] and CONSORT (Consolidated Standards of Reporting Trials) guidelines [31]. Figure 1 provides a flowchart of the study process.

### Study Setting

This study will be conducted at 2 PHC centers located in La Pintana and Puente Alto, 2 counties in Santiago, Chile, with low socioeconomic status. These counties have higher levels of poverty, overcrowding, and illiteracy than the national average.

### Eligibility Criteria

The population eligible for this study will be mothers with infants between 6 and 12 months of age attending either of the participating PHC centers. This age group was chosen because in the meta-analysis of the effectiveness of interventions to improve maternal sensitivity by Bakersman-Kranenburg et al [14], better results were observed with infants aged between 6 and 12 months than those under 6 months of age.

### Inclusion Criteria

The inclusion criteria are mothers of infants between 6 and 12 months who (1) attend 1 of the participating PHC centers, (2) are older than 18 years, (3) can speak Spanish fluently, and (4) have an electronic device that allows videoconferencing (ie, computer, tablet, or smart cell phone). In the case that a participant does not have internet access at home, we will consider supplying them with a prepaid card with internet gigabytes.

### Exclusion Criteria

The exclusion criteria are mothers with severe intellectual deficits or current psychotic symptoms and those participating in another early intervention at the PHC center.

### Interventions

#### Control Arm (Treatment as Usual Plus Educational Brochures)

Participants of this study are patients of the Chilean public health system and are supported by the Chile Crece Contigo national comprehensive child protection system. Some of the benefits are health checks during pregnancy, care during labor and delivery, child health checks, delivery of material for early stimulation, and detection and timely treatment of developmental delays [26].

In addition to receiving treatment as usual (TAU) at their PHC center, the control group will receive digital brochures with information on early parenting (once a week for 4 weeks). These brochures will summarize the content of each workshop session and the homework to be complete as a family. These are the same brochures handed out after each session in the intervention group.

#### Intervention Arm (TAU Plus C@nnected Group Videoconferencing Intervention)

The intervention group will participate in C@nnected, a group videoconferencing intervention adapted to the e-mental health format from a face-to-face brief attachment–based intervention that was previously designed, piloted [27], and evaluated through a randomized controlled trial (RCT) (results not yet published) to promote maternal sensitivity in mother-child dyads attending PHC centers. The face-to-face intervention was developed in 2012 following the framework proposed by the UK Medical Research Council for the development and evaluation of complex interventions [32] by the first 2 authors (VB and MO) of this study, considering the available evidence and local qualitative information. The core components obtained from the evidence were recommendations of the systematic review by Bakersman-Kranenburg et al [14] on effective interventions to improve parental sensitivity. The review shows that interventions that are carried out after 6 months of life, are of brief duration, and incorporate a second caregiver are more effective. From local qualitative research obtained through focus groups with both the possible users and providers of the intervention, different topics considered relevant by the participants and ideas on the best way to deliver the intervention were incorporated.

The general objective of the intervention is to enhance maternal sensitivity and promote skills in reading infant cues and responding sensitively, given that the inclusion of this variable in interventions, specifically in conditions of vulnerability, is associated with favorable results [33]. Each intervention session defines themes and objectives, worked through experiential activities, and immediately puts into practice the presented topics. In 1 of the sessions, another primary caregiver is actively invited to participate. At the end of each session, educational brochures with key concepts and homework are handed out to the participants to share and practice the new skills at home with the rest of the family**.**

The intervention is aimed at mother-infant dyads and consists of 4 sessions delivered over the course of 4 weeks, at 2 hours per week. Each group will include a minimum of 3 and a maximum of 6 dyads per group led by a trained provider. The activities are protocolized in a manual and are designed to be carried out with the infant. The manual specifies the structure and content of each session, the details of the materials to be used, relevant aspects to be emphasized during the activities, and additional information that delves more deeply into the issues addressed in the workshop. This aims to ensure the comprehensive replication of the intervention, which has been associated with the best effectiveness in this type of intervention [34]. Table 1 shows the main components of the intervention.

The adaptation to the video conferencing format was carried out between May and July 2021 by 2 authors of the face-to-face intervention in conjunction with a designer experienced with digital interventions. We used the recommendations of the Early Intervention Foundation [21] to achieve good results in virtual interventions as a frame of reference, which include the use of engagement elements and mechanisms to improve adherence and flexibility.

**Figure 2:**
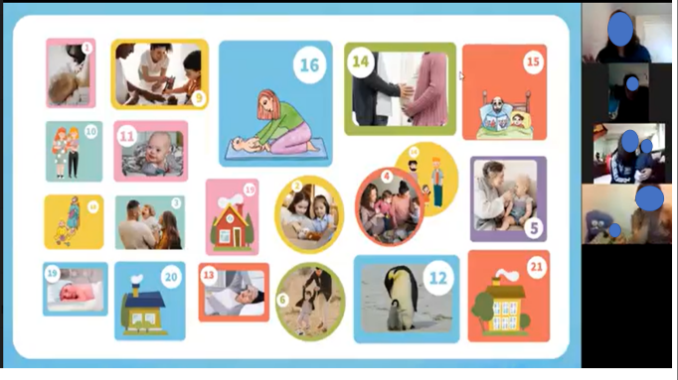
Screenshot of a videoconference session.

**Figure 3:**
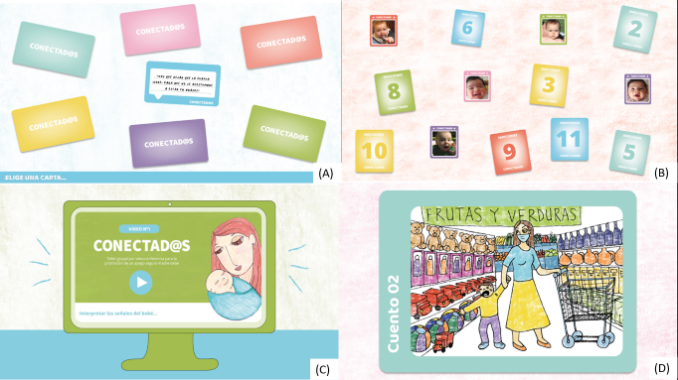
Screenshots illustrating different intervention activities. (A) Common myths in attachment and parenting; (B) working with different emotions and expressions in infants; (C) watching videos showing different parenting situations; (D) inventing a story from an image that shows the challenges of parenting.

We reviewed the complete content of the workshop, adjusted the sessions to 1.5 hours, and adapted the training manual and all the activities for use in a web-based group environment. We aim to provide lighthearted and interactive activities to allow all participants to understand and share their experiences. In addition, we have included some activities associated with the impact of the COVID- 19 pandemic. Figure 2 is a screenshot showing an example of a videoconference session, and Figure 3 shows 4 examples of activities that will take place in the workshop.

**Table 1:** Main components of the intervention with the objectives and activities of each session outlined.

Session	Name	Objectives	Examples of activities
1	“Knowing each other around attachment”	Achieve group cohesion Provide knowledge about attachment Recognize expected affective behaviors in an infant	Dynamics of presentation Difficulties in motherhood Common myths on attachment and parenting
2	“What does my baby need?”	Recognize crying as a signal of communication Recognize different expressions of emotions in babies Understand importance of responding to infant's needs	Recognize sensitive responses in caregivers Work with different emotions and expressions in infants
3	“Massages and agreements in parenting style” (Another relevant caregiver is invited)	Work on parental sensitivity through massage Involve another caregiver in the concept of attachment Achieve consensus among caregivers in some relevant issues of parenting	Infant massage with emphasis on sensitive interaction Watch locally made videos showing different parenting situations (eg, infant´s signs, safe exploration, talking and reading to children, and challenges in parenting)
4	“Boundaries and positive parenting”	Understand the importance of setting boundaries with respect and love Have strategies to set boundaries Resolve doubts regarding child abuse Recognize behaviors associated with positive parenting Know children's rights	Invent a story from an image that shows a difficulty in parenting Comment on experiences related to child abuse Express any doubts regarding the proper way to treat children Provide reflections on children's rights

In this study, each workshop will be led by a provider who is also a psychologist. Before conducting the intervention, the providers will receive a half-day training from the authors of the intervention. The video conferences will be recorded to guarantee the fidelity of the intervention and used later to perform a qualitative analysis of the change process [35].

To ensure internet connection during the evaluations and videoconferencing sessions, participants who do not have regular internet access will receive a prepaid card with internet gigabytes. To improve adherence to video conferencing sessions, the participating mothers will agree on the most convenient day and time for them to be held. In addition, a telephone chat group will be created to remind participants of the upcoming session and homework 1 day prior. If a participant cannot attend a videoconference session, they will be contacted by telephone by the provider to summarize the most important information of the session. This call will last between 15 and 20 minutes, and the main ideas and content of the session will be discussed, reinforcing the importance of positive interactions between mother and baby, sensitive care, and participation in the subsequent group sessions. For each participant to be considered to have received the intervention, they must attend at least 2 group videoconference sessions and 2 telephone calls or attend at least 3 of the 4 group video conference sessions.

### Outcome Measures

#### Primary Outcome Measures

The primary outcome measures are listed as follows.

#### Feasibility of the Intervention

Feasibility will be evaluated in terms of the following factors: (1) eligibility rates, referring to the proportion of mothers who meet inclusion criteria compared with the total number of mothers contacted by telephone; (2) recruitment rates, meaning the proportion of mothers who accept the invitation to participate in the study with respect to those who meet eligibility criteria; (3) adherence to group intervention, meaning the proportion of participating mothers in the intervention arm that receive the intervention (at least 2 group video conference sessions plus 2 telephone calls) and the average number of online sessions attended; and (4) follow-up rates by treatment condition, which refers to the proportion of participants that completed the postintervention assessment

#### Acceptability of the Intervention (Quantitative Assessment)

Satisfaction with the intervention will be measured with the Spanish version of the Credibility/Expectancy Questionnaire (CEQ) [36] at the preintervention assessment (t0) and 2 to 3 months later in the postintervention assessment (t1) for both arms of the study. This is a self-report instrument that comprises 6 items scored on a 9-point Likert-type scale ranging from 1 (not at all) to 9 (very) as well as 2 factors (credibility and expectation) that explain 82.46% of the variance. In terms of reliability, the total questionnaire showed a Cronbach α of .8, and scores range from 0 to 100, with higher scores indicating higher acceptability of the intervention.

#### Acceptability of the Intervention (Qualitative Assessment)

This measure will evaluate the experience of all participants of the intervention, both those providing the intervention and the mothers participating in the workshops, to determine which factors are associated with acceptability, barriers, and facilitators to implementation. This will be carried out as follows:

First, semistructured interviews will be conducted with the providers of the intervention. These interviews will inquire about the changes, achievements, and learning observed in the participants, the most and least valued topics and methodologies, difficulties in implementation and how it could be integrated into standard practice, possible improvements to the intervention, and core components of the intervention.

Second, focus group interviews with mothers participating in the intervention will be carried out. Two focus groups, each including 4 to 6 mothers, will be asked about their opinions regarding the usefulness of the intervention, changes, achievements, learning experiences, satisfaction, most and least valued topics and methodologies, and possible improvements. The aim of this focus group interview is to collect information on participants’ experience and identify possible improvements and the core components of the intervention.

#### Secondary Outcome Measures

**Figure 4:**
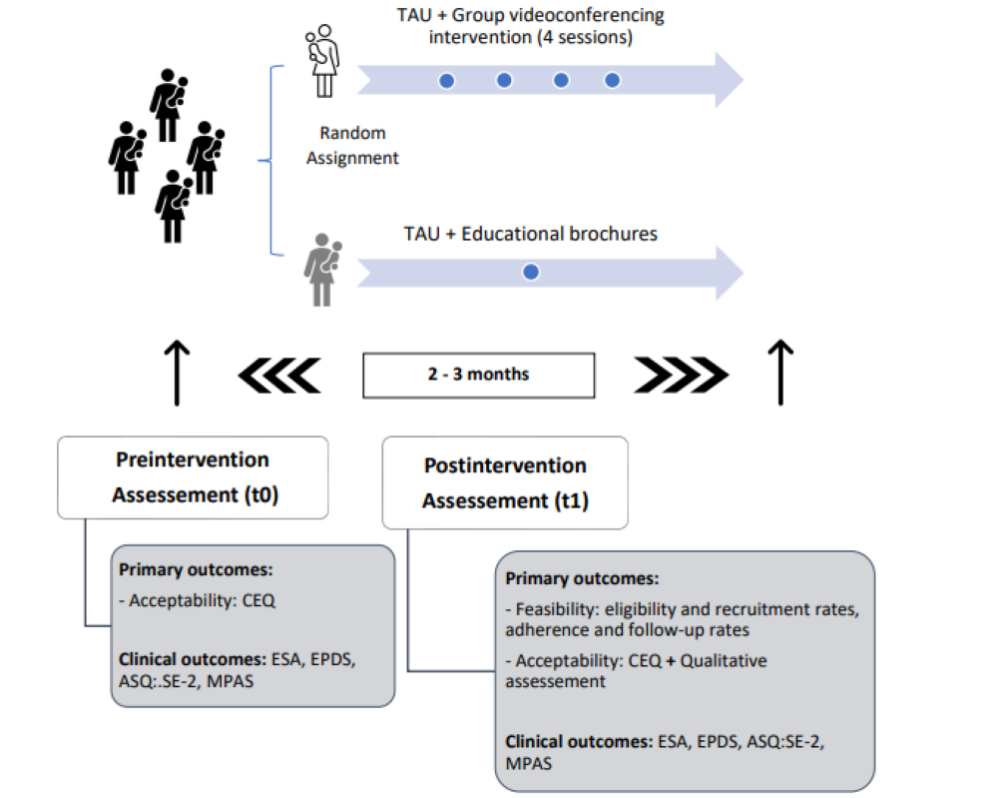
Study design schema. ASQ-SE-2: ages and stages questionnaire, social -emoaitonal; CEQ: measurement of credibility/expectancy questionnaire; ESA: adult sensibility scale; EPDS: Edinburgh postnatal depression scale; MPAS: maternal postnatal attachment scale; TAU: treatment as usual.

Clinical outcomes measures will be carried out to characterize the sample and estimate how the intervention could guide the sample size of the next effectiveness study. These clinical outcome measures will be assessed at the preintervention assessment (t0) and 2 to 3 months later in the postintervention assessment (t1). Table 2 shows the schedule of enrollment, interventions, and assessments, and Figure 4 shows the study design schema.

**Table 2:** Schedule of enrollment, interventions, and assessments.

Scheduled items		Study period				
		Enrollment	Preintervention assessment	Allocation	Intervention	Postintervention assessment
Time point		t0	t0			t1
**Enrollment**						
	Eligibility screen	✓				
	Informed consent	✓				
	Initial evaluation		✓			
	Allocation			✓		
**Interventions**						
	Intervention group (TAU^a^ + group videoconference intervention)				✓	
	Control group (TAU + educational brochures)				✓	
**Assessments**						
	Feasibility	✓	✓		✓	✓
	Acceptability: quantitative evaluation (CEQ^b^)		✓			✓
	Acceptability: qualitative evaluation					✓
	Clinical outcomes (ESA^c^, EPDS^d^, ASQ:SE-2^e^, MPAS^f^)		✓			✓

^a^TAU: treatment as usual.

^b^CEQ: Credibility/ Expectancy Questionnaire.

^c^ESA: Escala de Sensibilidad del Adulto (Adult Sensibility Scale).

^d^EPDS: Edinburgh Postnatal Depression Scale.

^e^ASQ:SE-2: Ages and Stages Questionnaire: Social-Emotional.

^f^MPAS: Maternal Postnatal Attachment Scale.

The secondary outcome measures are listed as follows.

#### Changes in Maternal Sensitivity

Changes in maternal sensitivity will be measured with the Escala de Sensibilidad del Adulto (ESA), also known as the Adult Sensitivity Scale [37]. Maternal sensitivity is the primary outcome that will also be measured in the effectiveness study since the objective of the intervention is to improve maternal sensitivity. This scale assesses an adult’s sensitivity in their interaction with children between 6 and 36 months of age. The mother and child are filmed for 5 minutes during free play interaction. The only instruction given is “do what you always do.” The coding system considers a rubric with 19 indicators that are related to different aspects of the sensitivity response. Each indicator is given a score between 1 and 3, with a higher score indicating higher sensitivity. In terms of reliability, the Cronbach α of the instrument is .93. The coders will be external to the research and blind to the randomized group, sociodemographic variables, and results of the different measurements. The Cohen kappa coefficient between the encoders will be obtained for the first 20 videos.

##### Changes in Maternal Depressive Symptoms

The measurement of postpartum depressive symptoms is of great relevance given its important association with low maternal sensitivity. This will be measured with the Edinburgh Postnatal Depression Scale (EPDS) [38], which is globally used to screen for maternal depression [39]. It consists of a self-administered questionnaire containing 10 multiple-choice questions, with 30 points as the maximum score and higher scores indicating higher depressive symptomatology. The scale has been validated in Chile [40] and has a sensitivity of 100%, a specificity of 80%, and adequate internal consistency (Cronbach α=.77). We will use the cutoff point of ≥10 to consider the presence of postpartum depressive symptoms.

#### Changes in Infants’ Socioemotional Development

The measurement of socioemotional development will allow for a more direct evaluation of the effects of the intervention on the participating infants. These will be measured with the Spanish version of the Ages and Stages Questionnaires: Social-Emotional (ASQ:SE-2) [41]. Several studies have consistently supported the precision and ease of use of ASQ-SE-2, which has been used widely in early mental health intervention programs [42]. We will use the 6- and 12-month versions. A higher score indicates worse socioemotional development. Each version has its specific cutoff score, and those with scores higher than the cutoff are considered to be “at risk for socioemotional development delay.”

#### Changes in Postpartum Maternal Bonding

The measurement of maternal bonding will allow us to chracterize the sample and evaluate possible changes to this parameter due to the intervention. We will use the Spanish version of the Maternal Postnatal Attachment Scale (MPAS) [43], a self-report measure that has 19 items ranging from 1 (low bonding) to 5 (high bonding) and is divided into 3 factors: quality of bonding, absence of hostility, and pleasure in interaction. Psychometric properties of the original version of the scale have shown an adequate internal consistency (Cronbach α=.78).

### Participant Timeline

The study duration for the participants will be 3 months, regardless of study arm allocation. This study does not have a follow-up phase. The total trial data collection period will be approximately 14 months.

### Sample Size

As this is a feasibility study, no hypotheses will be tested, and a formal power calculation is not required [44,45]. However, the evidence suggests a sample size of 25 people per arm for a small effect size of 0.2, which is suitable for clinical feasibility trials [45]. Therefore, a sample size of 50 participants was established, and the 3:2 allocation was chosen considering possible partial adherence to the intervention.

### Recruitment

Potential study participants will be identified from a list provided by each PHC center. Two research assistants (Rs), both clinical psychologists, will be in charge of calling the mothers included in the list, verify by telephone whether they meet the inclusion criteria, and explain the study in detail. Mothers who meet the inclusion criteria and are interested in participating will be sent an online protocol that will include informed consent. Mothers who do not wish to participate will be asked for the reasons. Once the mother provides electronic consent, the initial evaluation scales (EPDS, ASQ:SE- 2, MPAS, and CEQ) and a sociodemographic survey will be sent to her electronically. Finally, the mother and RA will agree upon at a time to make a video call to record 5 minutes of free play required by the ESA.

### Assignment of Interventions

#### Sequence Generation

To generate the random allocation sequence, we will use a computer software program called Studyrandomizer using permuted block randomization with a block size of 5 in a 3:2 ratio.

#### Concealment Mechanism

Allocation concealment will be ensured because the randomization code will not be released until all patients have been recruited and all initial measurements have been completed.

#### Implementation

After each participant signs the informed consent and performs baseline measurements, the RA recruiting the participant will contact another investigator to request the arm to which the participant has been randomized (intervention or intervention plus TAU). Thus, the enrollment of the participants will be performed by a different RA from the one who will generate the randomization sequence. The investigator will not be aware of the results of the initial evaluation. After completing the initial evaluation, the RA, who is blind to the random allocation sequence, will notify the participants of the type of intervention to which they will be assigned.

#### Blinding

Trial participants will be blinded to the conditions of the 2 arms; they will know the type of intervention to which they are assigned, but they will not know which of the interventions is considered the control. The intervention providers will be instructed not to disclose the treatment that participants are receiving. Follow-up measurements will be taken blindly for the assigned group. The assessments of outcome variables will be conducted by RAs who are blind to the treatment allocation.

#### Harms

No harm or risk to participants from this study is expected to occur. In cases where any alteration is detected in the postpartum depression and socioemotional development assessment scales, the mother will be informed and, with her consent, referred for management at her PHC center.

### Data Collection

Data obtained from the sociodemographic survey and the quantitative evaluations will be stored electronically and will not be linked to the identities of the participants. The data will be in a different spreadsheet than the one with personal data, which only 1 RA has access to, thus ensuring that the identity of the participants is protected.

Once the intervention is complete, both groups will complete the scales again (EPDS, ASQ:SE- 2, MPAS, and CEQ), and a new video recording (ESA) will be made by an RA different from the one who provided the intervention. Finally, the qualitative evaluation of the process will be carried out.

Data obtained from the qualitative information (the interviews and focus groups) will be recorded, transcribed, and assigned codes that will ensure the anonymization and protection of the participants’ identities.

### Data Analysis

For the quantitative analysis, descriptive statistics will be used for the clinical and sociodemographic variables of the groups, eligibility rate, recruitment, and adherence. Analysis of covariance (ANCOVA) will be used to determine differences in clinical outcomes between the groups. Data analysis will be conducted using SPSS Statistics 27 software (IBM Corp).

For the qualitative analysis, interviews and focus groups will be analyzed considering the conceptual basis of the Framework Analysis Approach [46], which is commonly used for thematic analysis of semistructured interview transcripts. It allows for the identification of commonalities and differences in qualitative data to draw descriptive and explanatory conclusions grouped around themes. It is different from Grounded Theory, which involves making systematic comparisons between cases to refine each topic and is aimed at generating social theory [47]. The Framework Analysis Approach is best suited to research that has specific questions, a limited time frame, a predesigned sample (eg, mothers and professionals participating in the intervention), and a priori issues (eg, evaluation of implementation of an intervention)

### Ethics Approval

All procedures and informed consent were approved by the Scientific Ethics Committee of Health Science of Pontificia Universidad Católica de Chile. The study was approved on March 11, 2021, and renewed on January 6, 2022, with validity of 1 year (# 200813008).

### Incentives

The participants will not receive any incentive for participating in this study. An incentive equivalent to US $12 will be offered to participants who complete the postintervention assessment.

## Results

The adaptation of the face-to-face intervention to the eHealth format was carried out between March and July 2021, and enrollment began in August 2021. Enrollment and data collection will continue until 50 participants have been enrolled and their data collected. We expect to complete the enrollment in August 2022 and for the primary impact analysis to be conducted in December 2022.

## Discussion

### Expected Findings

The objective of this study is to assess the feasibility and acceptability of the C@nnected group videoconferencing intervention, which aims to improve maternal sensitivity in mother-infant dyads attending PHC centers in Chile and was adapted from a face-to-face intervention. We expect to find an adequate feasibility of this intervention in terms of achieving good recruitment rates (around 60% mothers will accept the invitation to participate in the study with respect to those who meet eligibility criteria), good adherence to group intervention (around 60% in the intervention arm will receive the intervention), and adequate adherence to the sessions (the average expected attendance is 3 out of 4 sessions). We expect to find a very good acceptability of the intervention with high scores in the satisfaction questionnaire and positive evaluation in the qualitative assessment. We also expect to find favorable results of the intervention in terms of clinical outcomes, especially relating to maternal sensitivity, which will allow us to estimate the sample size for the future RCT to evaluate effectiveness. The results of this study will inform the key parameters for the implementation and evaluation of the intervention and facilitate the design of an effectiveness study in the future.

The COVID- 19 pandemic has led health teams to adapt some interventions to the eHealth format, and it is likely that many of these interventions will remain in this format even after the pandemic is over. Nevertheless, we should not assume that interventions will work equally as well when delivered through virtual methods. Existing interventions must be carefully adapted and include a focus on identifying the core components that must be maintained [21].

Internationally, there are some group parenting interventions with proven efficacy to increase maternal sensitivity; however, one of the main problems of this type of intervention is low adherence [48]. The development of eHealth interventions may improve the accessibility and flexibility of group-based interventions, which are important for parents. If this group intervention delivered by videoconference is shown to be feasible, it will be particularly useful for mothers that face barriers to attending face-to-face interventions.

### Limitations

This is a small pilot study in a highly vulnerable area of Santiago, Chile, so its results cannot necessarily be extrapolated to other contexts.

### Strengths

The main strengths of this study are as follows: 1) the intervention has proven effectiveness and was designed locally using a framework that was later adapted for the electronic format; 2) it will evaluate the acceptability and feasibility of the intervention both quantitatively and quantitatively; and 3) it will evaluate clinical outcomes. In our opinion, these are essential steps to take before conducting a randomized controlled study, and they will subsequently allow us to scale up this intervention in similar contexts once its effectiveness is evaluated.

### Conclusions

This study will lay the foundation for a randomized clinical trial to examine the effectiveness of an intervention to improve maternal sensitivity in mothers attending PHC centers.

## Abbreviations

ANCOVA: analysis of covariance
ASQ: SE- 2: Ages and Stages Questionnaires: Social-Emotional
CEQ: Credibility/ Expectancy Questionnaire
CONSORT: Consolidated Standards of Reporting Trials
EPDS: Edinburgh Postnatal Depression Scale
ESA: Escala de Sensibilidad del Adulto
MIDAP: Millennium Institute for Research on Depression and Personality
MPAS: Maternal Postnatal Attachment Scale
PHC: primary health care
RA: research assistant
RCT: randomized controlled trial
SPIRIT: Standard Protocol Items: Recommendations for Interventional Trials
TAU: treatment as usual

## Data Availability

In keeping with transparency recommendations, data will be made publicly available after primary analyses in a public data repository. To promote dissemination of the study findings to potential end users, patients, and clinicians, as well as the scientific community, the findings will be published in academic journals presented at conferences. Primary health care center directors will also receive the manuscripts.
